# Supplementary material for: Epigenome-wide DNA methylation association study of CHIP provides insight into perturbed gene regulation
Source: Nat Commun. 2025 May 20;16:4678. doi: 10.1038/s41467-025-59333-w (PMC12092741; doi:10.1038/s41467-025-59333-w)
Supplement: Supplementary file 1 — Supplementary Information File [file 41467_2025_59333_MOESM1_ESM.docx]

**Epigenome-wide DNA Methylation Association Study of CHIP Provides Insight into Perturbed Gene Regulation**

**Supplementary Methods**

Cohort Descriptions

Framingham Heart Study (FHS)

DNA methylation measurements and quality control:

DNA was extracted from whole blood buffy coat samples using Gentra Puregene DNA extraction kit (Qiagen, Venlo, Netherland). Samples subsequently underwent bisulfite conversion via the EZ DNA methylation kit (Zymo Research, Irvine, CA), whole genome amplification, fragmentation, array hybridization, and single base pair extension. DNA methylation was measured using the Ilumina Infinium Human Methylation-450 Beadchip (450K array) (Ilumina Inc., San Diego, CA). DNA samples from the FHS offspring cohort were run in two laboratory batches: Johns Hopkins Center for Inherited Disease Research (batch #1) and University of Minnesota Biomedical Genomic Center (batch #2). DNA methylation arrays of Third Generation cohort samples (batch #3) were run by Ilumina (San Diego, CA, USA). For each batch, DNA methylation β values were normalized using the DASEN methodology in the wateRmelon R package, and the output β values for each CpG were used in analysis.^1^ DNA methylation β value is indicative of the proportion of methylated DNA at a CpG site in a sample, ranging from 0 (unmethylated) to 1 (fully methylated).

For quality control (QC), samples were excluded with a missing β value (detection P > 0.01) at >1% CpG sites, poor matching of single nucleotide polymorphisms (SNPs) between the 65 SNPs on the Ilumina 450K array and GWAS array, or were outliers in the multi-dimensional scaling plot. 4170 samples – 2648 Offspring Generation cohort samples and 1522 Third Generation cohort samples – passed final QC. Probes were excluded with missing β values (detection P > 0.01) at >20% samples, that were previously mapped to multiple locations on sex chromosomes,^2^ or had an underlying SNP (minor allele frequency [MAF] >5% in EA 1000 Genomes Project data) at the CpG site or within 10 bp of the single base pair extension.^3^ 415,318 CpGs were ultimately retained for analysis.

Epigenome-wide association study of CHIP:

Surrogate variable analyses (SVA)^4^ were used to eliminate hidden, unwanted variation in DNA methylation data. SVs were generated in each batch separately; DNA methylation β values were regressed on batch-specific SVs, and the DNAm residual was taken forward. The two batches were merged together for analysis. Linear mixed models were used to test associations between CHIP status as the predictor variable and DNA methylation β value, adjusted by SVs, as the outcome variable. Covariates including age, age^2^, sex, smoking, technical confounders, cell types imputed by Houseman method^5^ and familial relatedness were further included in the model.

Jackson Heart Study (JHS)

DNA methylation measurements and quality control:

DNA was extracted from whole blood samples from the JHS baseline examination, and DNA methylation was measured using the Ilumina EPIC (850K) array, as previously described. The majority of methylation data was from Visit 1, with a subset from Visit 2. DNA methylation data were normalized using the minfi R package.^6, 7^ Noob normalization and generation of methylation beta values was performed by Steve Horvath’s lab.^8, 9^ Outlier samples (N=46) were identified based on hierarchical clustering and removed. Potentially cross-reactive CpGs sites and sites near common African American polymorphisms were removed.

Epigenome-wide association study of CHIP:

Initially, DNA methylation beta values underwent adjustment for known batch effects using the COMBat method,^10^ with the residuals retained for subsequent analysis. Linear mixed models were then employed to investigate associations between CHIP status, serving as the predictor variable, and DNA methylation residuals (post-COMBat adjustment), serving as the outcome variable. Additionally, adjustments were made for standard covariates, including age, age^2^, sex, smoking status, and cell type proportions encompassing CD8T, CD4T, NK cells, B cells, monocytes, and granulocytes. Estimates of cell type proportions for each sample were derived utilizing the Houseman method^5^ as implemented by Steve Horvath.

Atherosclerosis Risk In Communities (ARIC) Study

DNA methylation measurements and quality control:

DNA was extracted from peripheral blood leukocyte samples, and DNA methylation was measured using the Ilumina Infinium Human Methylation-450 Beadchip (450K array) (Ilumina Inc., San Diego, CA).

For quality control, samples were excluded if the pass rate was <95% (number of probes with a detection P < 0.01/number of probes on array), sex mismatch was likely based on principal component analysis, or genotypes for 24 SNPs on the 450K array were inconsistent with prior genotyping. CpG sites were excluded if >5% of samples had a detection P > 0.01, or if the average detection P for a Y chromosome CpG site was >0.01 among male participants. Beta Mixture Quantile dilation (BMIQ) method using Noob background and dye bias correction was implemented for normalizing DNA methylation values.^8, 11^

Epigenome-wide association study of CHIP:

Separate EWAS were performed in self-reported Black and White individuals. Linear models were used for each EWAS to test associations between CHIP status as the independent variable and DNA methylation proportion as the outcome variable, with adjustment for age, age^2^, sex, batch effects, and estimated cell type proportions.^12^

The Cardiovascular Health Study (CHS)

DNA methylation measurements and quality control:

DNA methylation was measured using Ilumina Infinium Human Methylation-450 Beadchip (450K array)

The minfi R package was used for DNA methylation QC.^6, 7^ Samples were excluded if median intensities across the methylated and unmethylated changes were <10.5 (log 2), >0.5% of probes failed detection, QC probes fell >3 SD from the mean, or sample swaps were likely due to sex mismatches or genotype inconsistency with prior genotyping. Subset-quantile Within Array Normalization (SWAN) was implemented to standardize DNA methylation values^13^, and the Houseman, et al. method was used to estimate blood cell proportions from DNA methylation data.^5^

Epigenome-wide association study of CHIP:

Separate EWAS were performed in self-reported Black and White individuals. Linear models were used for each EWAS to test associations between CHIP status as the independent variable and DNA methylation proportion as the outcome variable, with adjustment for age, age^2^, sex, batch effects, estimated cell type proportions, and individual random effects to account for repeated measurements from two timepoints.^12^

**Supplementary Figures**

c.

b.

a.

Any CHIP

*DNMT3A* CHIP

*TET2* CHIP


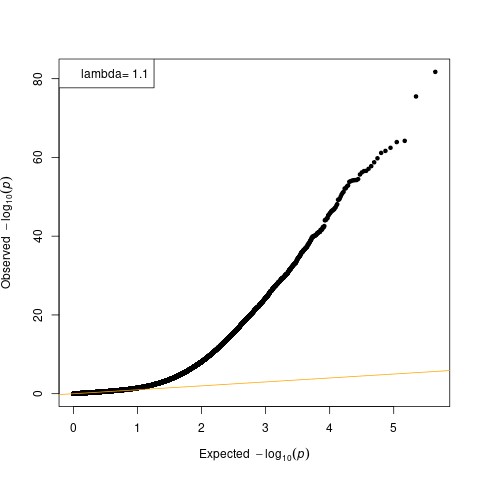

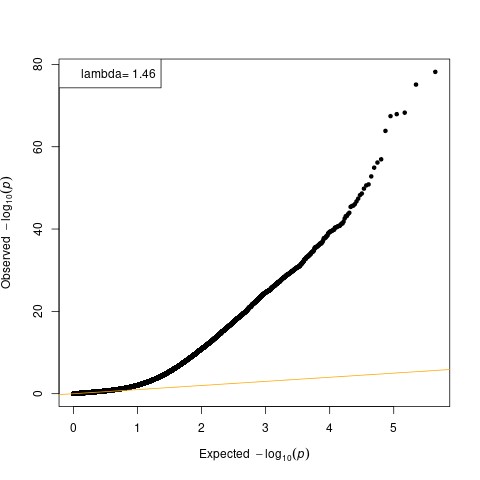

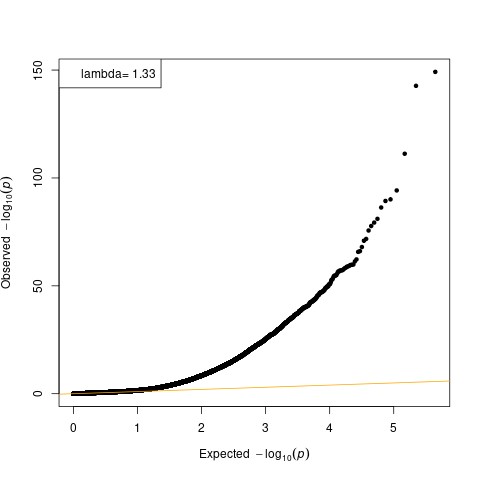


d.

*ASXL1* CHIP


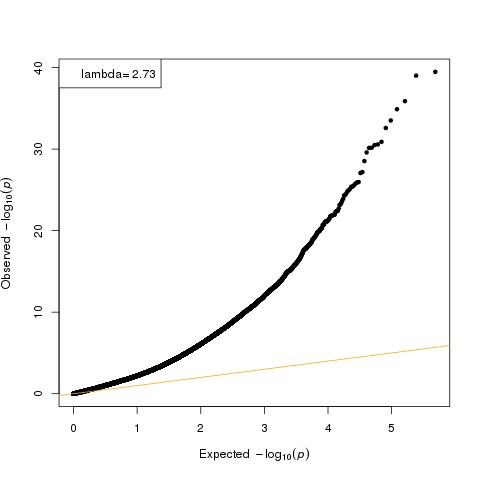


**Supplementary Figure 1: Quantile-quantile plots of expected and observed** $\boldsymbol{-log}_{\boldsymbol{10}}\boldsymbol{(P)}$ **in multiracial meta-EWAS of CHIP and CHIP subtypes.** Genomic inflation factors were computed for the meta-EWAS of any CHIP and CHIP subtypes. (a) λ = 1.46 for any CHIP, (b) λ = 1.33 for *DNMT3A* CHIP, (c) λ = 1.1 for *TET2* CHIP, and (d) λ = 2.73 for *ASXL1* CHIP. Source data are provided as a Source Data file.

a.

c.

b.


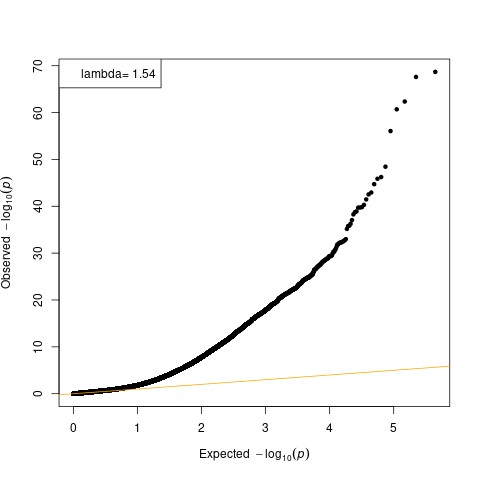

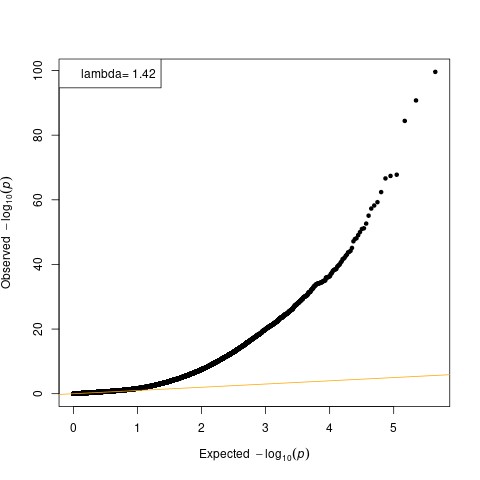

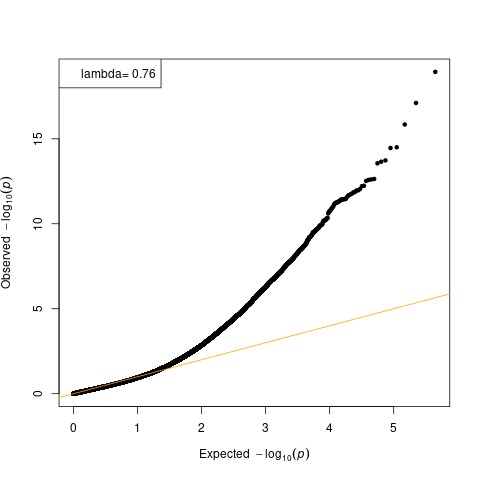


*TET2* CHIP

*DNMT3A* CHIP

Any CHIP

**Supplementary Figure 2. Quantile-quantile plots of expected and observed** $\boldsymbol{-log}_{\boldsymbol{10}}\boldsymbol{(P)}$ **in CHIP meta EWAS of Black participants.** Genomic inflation factors were computed for the meta-EWAS of any CHIP and CHIP subtypes in Black participants. (a) λ = 1.54 for any CHIP, (b) λ = 1.42 for *DNMT3A* CHIP, (c) λ = 0.76 for *TET2* CHIP. Source data are provided as a Source Data file.

Any CHIP

*DNMT3A* CHIP

*TET2* CHIP

c.

b.

a.


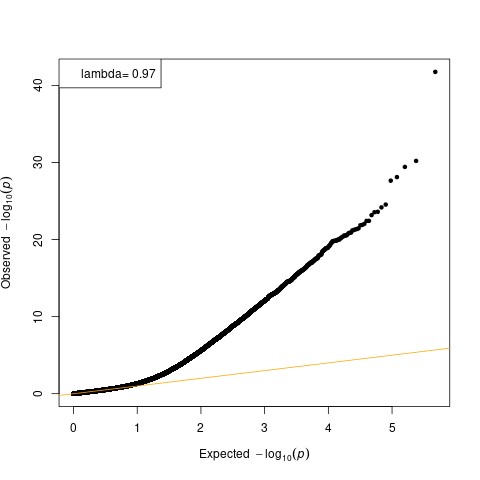

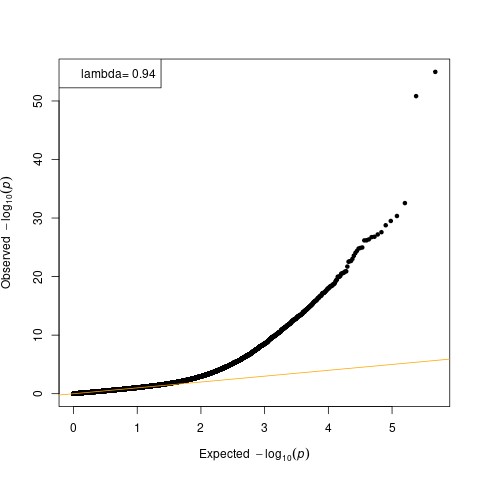

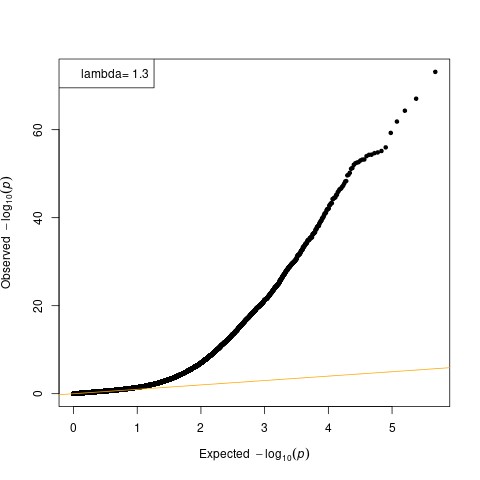


**Supplementary Figure 3. Quantile-quantile plots of expected and observed** $\boldsymbol{-log}_{\boldsymbol{10}}\boldsymbol{(P)}$ **in CHIP meta EWAS of White participants.** Genomic inflation factors were computed for the meta-EWAS of any CHIP and CHIP subtypes in White participants. (a) λ = 0.97 for any CHIP, (b) λ = 0.94 for *DNMT3A* CHIP, (c) λ = 1.3 for *TET2* CHIP. Source data are provided as a Source Data file.

b.

a.


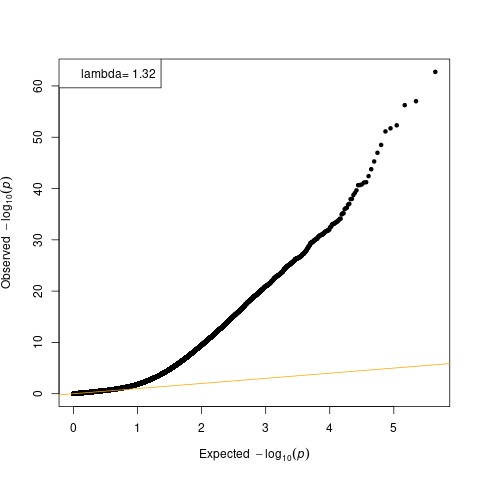


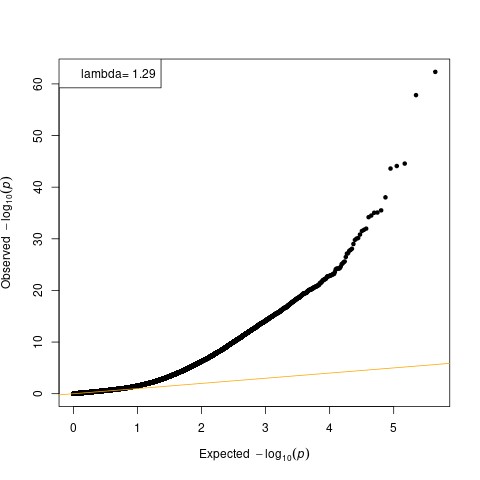


c.


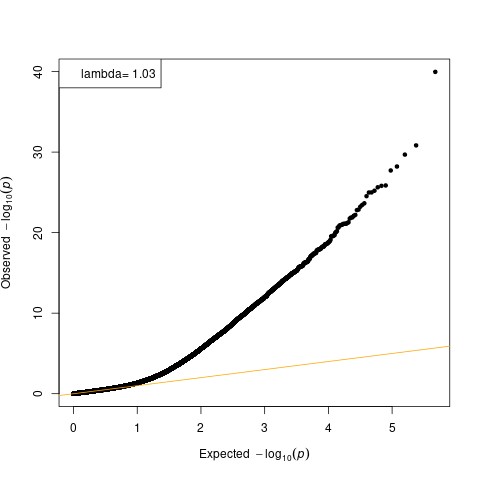


**Supplementary Figure 4. Quantile-quantile plots of expected and observed** $\boldsymbol{-log}_{\boldsymbol{10}}\boldsymbol{(P)}$ **for meta-EWAS of any CHIP with VAF > 10%.** Genomic inflation factors were computed for the meta-EWAS of any CHIP with VAF > 10% in (a) all races: λ = 1.32, (b) in Black participants: λ = 1.29, (c) in White participants: λ = 1.03. Source data are provided as a Source Data file.


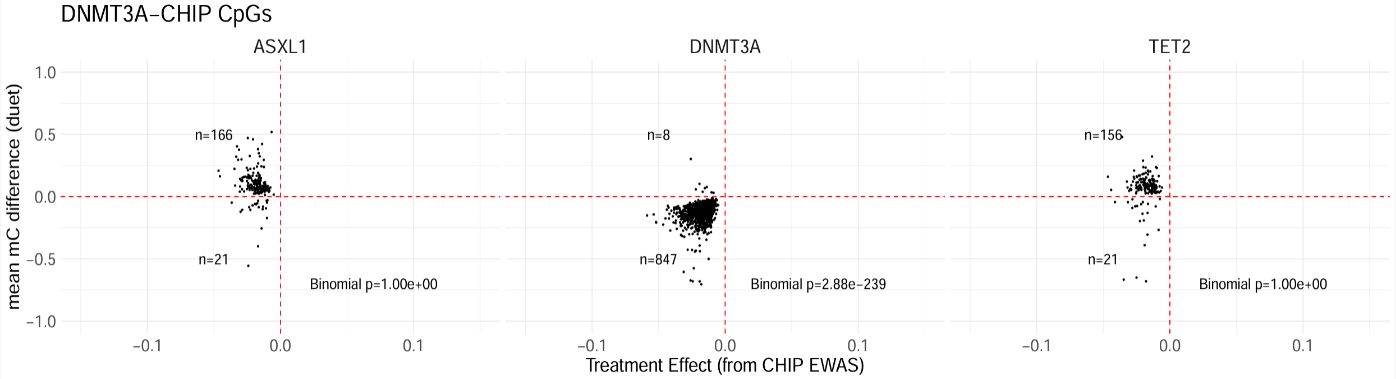

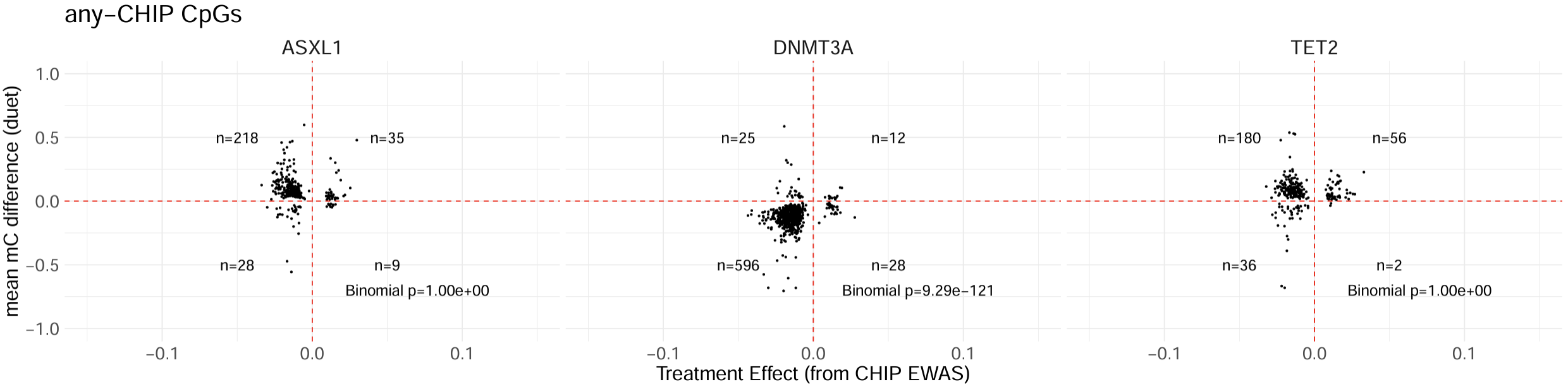

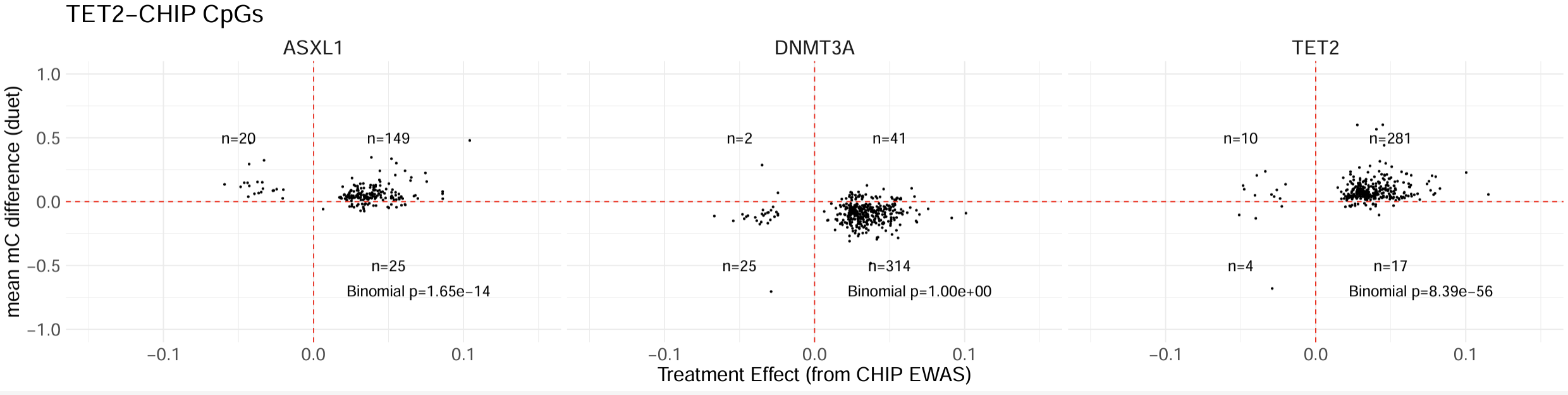

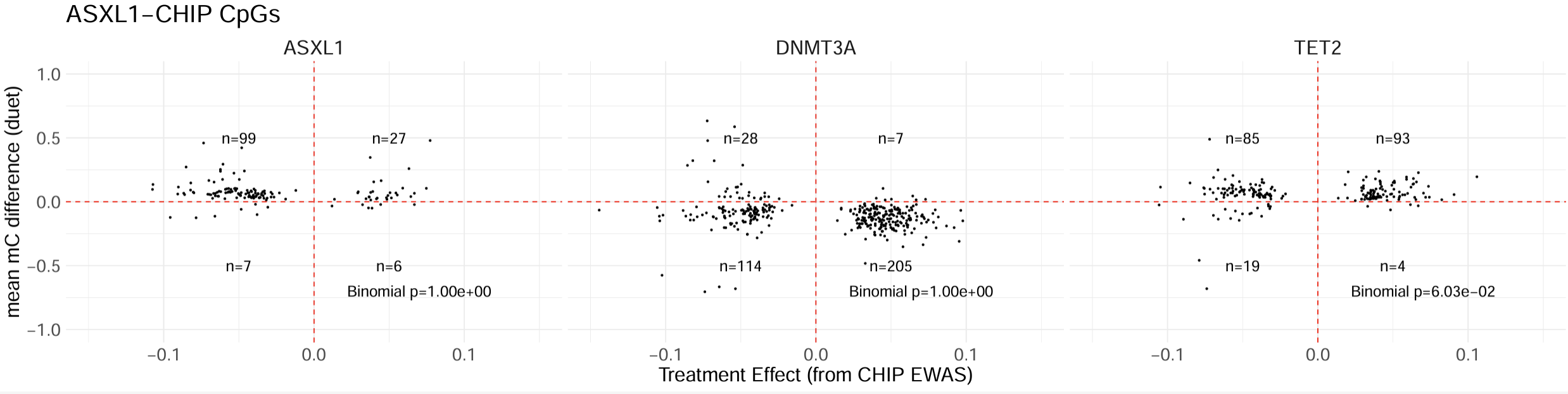


Supplementary Figure 5. **Correlation of CpG Sites in Engineered Human Stem Cells**

Dot plots of methylation change from -1.0 (no methylation) to 1.0 (complete methylation) seen in engineered primary cell cultures compared to correlation of EWAS results ranging from -0.1 to 0.1. Following an initial CpG filtering using an uncorrected Student’s t-test (p<0.05), significance was determined with a two-sided binomial test. a) Comparison of any CHIP CpG sites compared to each engineered cell line. b) Comparison of *DNMT3A*-CHIP CpG sites compared to each engineered cell culture. c) Comparison of *TET2*-CHIP CpG sites compared to each engineered cell culture. d) Comparison of *ASXL*-1 CHIP CpG site compared to each engineered cell culture. Source data are provided as a Source Data file.

a.

b.

c.

d.

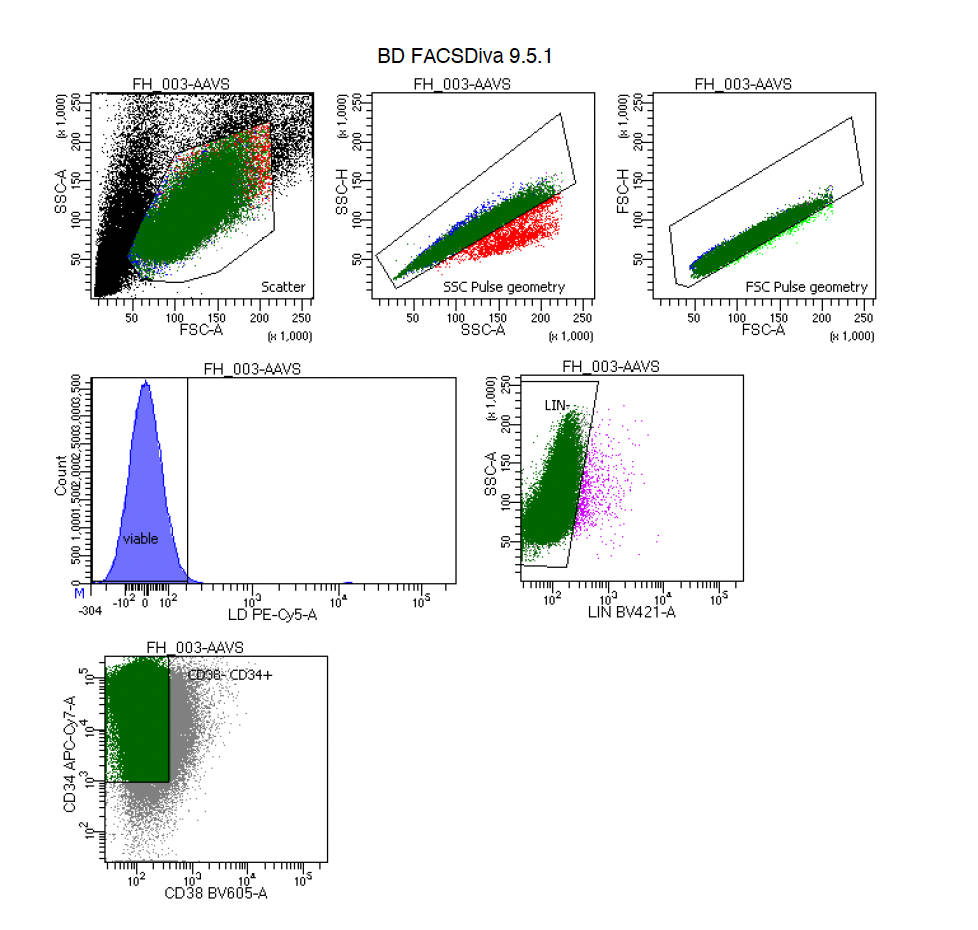


Supplementary Figure 6. **Representative Fluorescent Activated Cell** **Sorting**

Flow gating strategy for sorting human hematopoietic stem cells using 7-AAD (live/dead stain), CD34+ (APC-Cy7), CD38- (BV605), and Lineage- (Pacific Blue).

**Supplementary Tables**


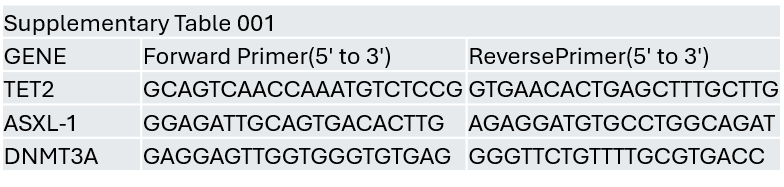


Supplementary Table 1. Primer sequences for assessing indel formation present in each sgRNA.


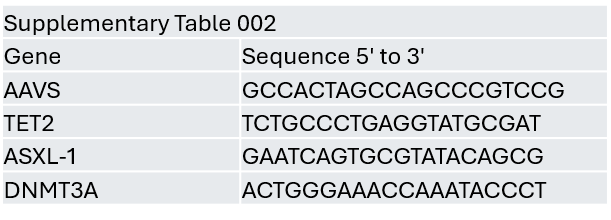


Supplementary Table 2. Single guide RNA sequences

SgRNA sequences for each guide RNA targeting AAVS, TET2, ASXL1, and DNMT3A.


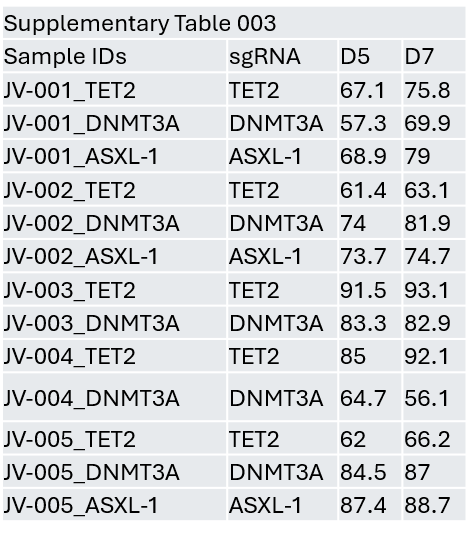


**
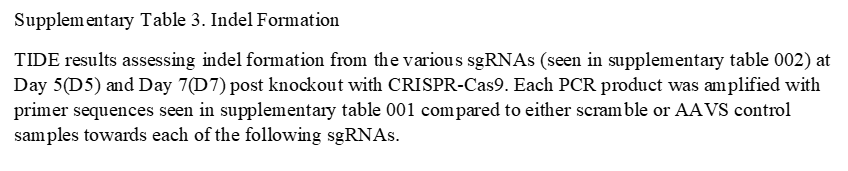
**

Supplementary Table 3. Indel Formation

TIDE results assessing indel formation from the various sgRNAs (seen in supplementary table 002) at Day 5(D5) and Day 7(D7) post knockout with CRISPR-Cas9. Each PCR product was amplified with primer sequences seen in supplementary table 001 compared to either scramble or AAVS control samples towards each of the following sgRNAs.

Supplementary Table 3. Indel Formation

TIDE results assessing indel formation from the various sgRNAs (seen in supplementary table 002) at Day 5(D5) and Day 7(D7) post knockout with CRISPR-Cas9. Each PCR product was amplified with primer sequences seen in supplementary table 001 compared to either scramble or AAVS control samples towards each of the following sgRNAs.

Supplementary Figure 9. Indel Formation

TIDE results assessing indel formation from the various sgRNAs (seen in supplementary table 002) at Day 5(D5) and Day 7(D7) post knockout with CRISPR-Cas9. Each PCR product was amplified with primer sequences seen in supplementary table 001 compared to either scramble or AAVS control samples towards each of the following sgRNAs.

Supplementary Figure 9. Indel Formation

TIDE results assessing indel formation from the various sgRNAs (seen in supplementary table 002) at Day 5(D5) and Day 7(D7) post knockout with CRISPR-Cas9. Each PCR product was amplified with primer sequences seen in supplementary table 001 compared to either scramble or AAVS control samples towards each of the following sgRNAs.

Supplementary Figure 9. Indel Formation

TIDE results assessing indel formation from the various sgRNAs (seen in supplementary table 002) at Day 5(D5) and Day 7(D7) post knockout with CRISPR-Cas9. Each PCR product was amplified with primer sequences seen in supplementary table 001 compared to either scramble or AAVS control samples towards each of the following sgRNAs.

Supplementary Figure 9. Indel Formation

TIDE results assessing indel formation from the various sgRNAs (seen in supplementary table 002) at Day 5(D5) and Day 7(D7) post knockout with CRISPR-Cas9. Each PCR product was amplified with primer sequences seen in supplementary table 001 compared to either scramble or AAVS control samples towards each of the following sgRNAs.

| Supplementary Table 004 |  |  |
| --- | --- | --- |
| Human Antibody \| Fluorophore | Catalog Number | Dilution |
| Hu CD34 \| APC-Cy7 | 343614 | 1:50 |
| Hu CD38 \| BV605 | 303532 | 1:100 |
| Hu Lineage Cocktail \| Pacific Blue | 348805 | 1:10 |

Supplementary Table 4. Human Antibodies

Human Antibodies used towards CD34, CD38 and Lineage cocktail all from Biolegend.

**Supplementary References**

1. Pidsley R, CC YW, Volta M, Lunnon K, Mill J, Schalkwyk LC. A data-driven approach to preprocessing Illumina 450K methylation array data. BMC Genomics. 2013;14:293. Epub 20130501. doi: 10.1186/1471-2164-14-293. PubMed PMID: 23631413; PMCID: PMC3769145.

2. Price ME, Cotton AM, Lam LL, Farre P, Emberly E, Brown CJ, Robinson WP, Kobor MS. Additional annotation enhances potential for biologically-relevant analysis of the Illumina Infinium HumanMethylation450 BeadChip array. Epigenetics Chromatin. 2013;6(1):4. Epub 20130303. doi: 10.1186/1756-8935-6-4. PubMed PMID: 23452981; PMCID: PMC3740789.

3. Chen YA, Lemire M, Choufani S, Butcher DT, Grafodatskaya D, Zanke BW, Gallinger S, Hudson TJ, Weksberg R. Discovery of cross-reactive probes and polymorphic CpGs in the Illumina Infinium HumanMethylation450 microarray. Epigenetics. 2013;8(2):203-9. Epub 20130111. doi: 10.4161/epi.23470. PubMed PMID: 23314698; PMCID: PMC3592906.

4. Leek JT, Storey JD. Capturing heterogeneity in gene expression studies by surrogate variable analysis. PLoS Genet. 2007;3(9):1724-35. Epub 20070801. doi: 10.1371/journal.pgen.0030161. PubMed PMID: 17907809; PMCID: PMC1994707.

5. Houseman EA, Accomando WP, Koestler DC, Christensen BC, Marsit CJ, Nelson HH, Wiencke JK, Kelsey KT. DNA methylation arrays as surrogate measures of cell mixture distribution. BMC Bioinformatics. 2012;13:86. Epub 20120508. doi: 10.1186/1471-2105-13-86. PubMed PMID: 22568884; PMCID: PMC3532182.

6. Aryee MJ, Jaffe AE, Corrada-Bravo H, Ladd-Acosta C, Feinberg AP, Hansen KD, Irizarry RA. Minfi: a flexible and comprehensive Bioconductor package for the analysis of Infinium DNA methylation microarrays. Bioinformatics. 2014;30(10):1363-9. Epub 20140128. doi: 10.1093/bioinformatics/btu049. PubMed PMID: 24478339; PMCID: PMC4016708.

7. Fortin JP, Labbe A, Lemire M, Zanke BW, Hudson TJ, Fertig EJ, Greenwood CM, Hansen KD. Functional normalization of 450k methylation array data improves replication in large cancer studies. Genome Biol. 2014;15(12):503. Epub 20141203. doi: 10.1186/s13059-014-0503-2. PubMed PMID: 25599564; PMCID: PMC4283580.

8. Triche TJ, Jr., Weisenberger DJ, Van Den Berg D, Laird PW, Siegmund KD. Low-level processing of Illumina Infinium DNA Methylation BeadArrays. Nucleic Acids Res. 2013;41(7):e90. Epub 20130309. doi: 10.1093/nar/gkt090. PubMed PMID: 23476028; PMCID: PMC3627582.

9. Teschendorff AE, Marabita F, Lechner M, Bartlett T, Tegner J, Gomez-Cabrero D, Beck S. A beta-mixture quantile normalization method for correcting probe design bias in Illumina Infinium 450 k DNA methylation data. Bioinformatics. 2013;29(2):189-96. Epub 20121121. doi: 10.1093/bioinformatics/bts680. PubMed PMID: 23175756; PMCID: PMC3546795.

10. Jiao C, Zhang C, Dai R, Xia Y, Wang K, Giase G, Chen C, Liu C. Positional effects revealed in Illumina methylation array and the impact on analysis. Epigenomics. 2018;10(5):643-59. Epub 20180222. doi: 10.2217/epi-2017-0105. PubMed PMID: 29469594; PMCID: PMC6021926.

11. Teschendorff AE, Marabita F, Lechner M, Bartlett T, Tegner J, Gomez-Cabrero D, Beck S. A beta-mixture quantile normalization method for correcting probe design bias in Illumina Infinium 450 k DNA methylation data. Bioinformatics. 2012;29(2):189-96.

12. Uddin MDM, Nguyen NQH, Yu B, Brody JA, Pampana A, Nakao T, Fornage M, Bressler J, Sotoodehnia N, Weinstock JS, Honigberg MC, Nachun D, Bhattacharya R, Griffin GK, Chander V, Gibbs RA, Rotter JI, Liu C, Baccarelli AA, Chasman DI, Whitsel EA, Kiel DP, Murabito JM, Boerwinkle E, Ebert BL, Jaiswal S, Floyd JS, Bick AG, Ballantyne CM, Psaty BM, Natarajan P, Conneely KN. Clonal hematopoiesis of indeterminate potential, DNA methylation, and risk for coronary artery disease. Nat Commun. 2022;13(1):5350. Epub 20220912. doi: 10.1038/s41467-022-33093-3. PubMed PMID: 36097025; PMCID: PMC9468335.

13. Maksimovic J, Gordon L, Oshlack A. SWAN: Subset-quantile within array normalization for illumina infinium HumanMethylation450 BeadChips. Genome Biol. 2012;13(6):R44. Epub 20120615. doi: 10.1186/gb-2012-13-6-r44. PubMed PMID: 22703947; PMCID: PMC3446316.
