# Supplementary material for: Epigenome-wide DNA methylation association study of CHIP provides insight into perturbed gene regulation
Source: Nat Commun. 2025 May 20;16:4678. doi: 10.1038/s41467-025-59333-w (PMC12092741; doi:10.1038/s41467-025-59333-w)
Supplement: Supplementary file 2 — Description of Additional Supplementary Files [file 41467_2025_59333_MOESM2_ESM.pdf]

Description of Additional Supplementary Files

File Name: Supplementary Data 1

Description: **Meta-EWAS of any CHIP in Black Participants.** A race-stratified meta-EWAS of any CHIP was performed in JHS, CHS, and ARIC. Significance was defined as a Bonferroni-corrected P-value < 1×10^{-7}, based on two-sided P-values from an inverse variance-weighted fixed-effect meta-analysis.

File Name: Supplementary Data 2

Description: **Meta-EWAS of *DNMT3A* CHIP in Black Participants.** A race-stratified meta-EWAS of any CHIP was performed in JHS, CHS, and ARIC. Significance was defined as a Bonferroni-corrected P-value < 1×10^{-7}, based on two-sided P-values from an inverse variance-weighted fixed-effect meta-analysis.

File Name: Supplementary Data 3

Description: **Meta-EWAS of *TET2* CHIP in Black Participants.** A race-stratified meta-EWAS of any CHIP was performed in JHS, CHS, and ARIC. Significance was defined as a Bonferroni-corrected P-value < 1×10^{-7}, based on two-sided P-values from an inverse variance-weighted fixed-effect meta-analysis.

File Name: Supplementary Data 4

Description: **Meta-EWAS of any CHIP in White Participants.** A race-stratified meta-EWAS of any CHIP was performed in FHS, CHS, and ARIC. Significance was defined as a Bonferroni-corrected P-value < 1×10^{-7}, based on two-sided P-values from an inverse variance-weighted fixed-effect meta-analysis.

File Name: Supplementary Data 5

Description: **Meta-EWAS of *DNMT3A* CHIP in White Participants.** A race-stratified meta-EWAS of any CHIP was performed in FHS, CHS, and ARIC. Significance was defined as a Bonferroni-corrected P-value < 1×10^{-7}, based on two-sided P-values from an inverse variance-weighted fixed-effect meta-analysis.

File Name: Supplementary Data 6

Description: **Meta-EWAS of *TET2* CHIP in White Participants.** A race-stratified meta-EWAS of any CHIP was performed in FHS, CHS, and ARIC. Significance was defined as a Bonferroni-corrected P-value < 1×10^{-7}, based on two-sided P-values from an inverse variance-weighted fixed-effect meta-analysis.

File Name: Supplementary Data 7

Description: **Multiracial Meta-EWAS of any CHIP.** A multi-ancestry meta-EWAS of any CHIP was performed in FHS, JHS, CHS, and ARIC. Significance was defined as a Bonferroni-corrected P-value < 1×10^{-7}, based on two-sided P-values from an inverse variance-weighted fixed-effect meta-analysis.

File Name: Supplementary Data 8

Description: **Multiracial Meta-EWAS of *DNMT3A* CHIP.** A multiracial meta-EWAS of any CHIP was performed in FHS, JHS, CHS, and ARIC. Significance was defined as a Bonferroni-corrected P-value < 1×10^{-7}, based on two-sided P-values from an inverse variance-weighted fixed-effect meta-analysis.

File Name: Supplementary Data 9

Description: **Multiracial Meta-EWAS of *TET2* CHIP.** A multiracial meta-EWAS of any CHIP was performed in FHS, JHS, CHS, and ARIC. Significance was defined as a Bonferroni-corrected P-value < 1×10^{-7}, based on two-sided P-values from an inverse variance-weighted fixed-effect meta-analysis.

File Name: Supplementary Data 10

Description: **EWAS of *ASXL1* CHIP.** A EWAS of *ASXL1* CHIP was performed exclusively in the FHS cohort. Significance was defined as a Bonferroni-corrected P-value < 1×10^{-7}, based on two-sided P-values from an inverse variance-weighted fixed-effect meta-analysis.

File Name: Supplementary Data 11

Description: **Meta-EWAS Summary Statistics for any CHIP with VAF > 10% in Black Participants.** Significance was defined as a Bonferroni-corrected P-value < 1×10^{-7}, based on two-sided P-values from an inverse variance-weighted fixed-effect meta-analysis.

File Name: Supplementary Data 12

Description: **Meta-EWAS Summary Statistics for any CHIP with VAF > 10% in White Participants.** Significance was defined as a Bonferroni-corrected P-value < 1×10^{-7}, based on two-sided P-values from an inverse variance-weighted fixed-effect meta-analysis.

File Name: Supplementary Data 13

Description: **Multiracial Meta-EWAS Summary Statistics for any CHIP with VAF > 10%.** Significance was defined as a Bonferroni-corrected P-value < 1×10^{-7}, based on two-sided P-values from an inverse variance-weighted fixed-effect meta-analysis.

File Name: Supplementary Data 14

Description: **Gene Ontology (GO) Enrichment Analysis for Genes Harboring any CHIP-associated CpGs.** The missMethyl R package was used for GO enrichment analysis. Fisher's Exact Test (one-sided) was used for this analysis.

File Name: Supplementary Data 15

Description: **Gene Ontology Enrichment Analysis for Genes Harboring *DNMT3A* CHIP-associated CpGs.** The missMethyl R package was used for GO enrichment analysis. Fisher's Exact Test (one-sided) was used for this analysis.

File Name: Supplementary Data 16

Description: **Gene Ontology Enrichment Analysis for Genes Harboring *TET2* CHIP-associated CpGs.** The missMethyl R package was used for GO enrichment analysis. Fisher's Exact Test (one-sided) was used for this analysis.

File Name: Supplementary Data 17

Description: **Gene Ontology Enrichment Analysis for Genes Harboring *ASXL1* CHIP-associated CpGs.** The missMethyl R package was used for GO enrichment analysis. Fisher's Exact Test (one-sided) was used for this analysis.

File Name: Supplementary Data 18

Description: **Any CHIP Expression Quantitative Trait Methylation Loci (eQTMs).** Linear regression (two-sided) was employed for this analysis.

File Name: Supplementary Data 19

Description: ***DNMT3A* CHIP Expression Quantitative Trait Methylation Loci (eQTMs).** Linear regression (two-sided) was employed for this analysis.

File Name: Supplementary Data 20

Description: ***TET2* CHIP Expression Quantitative Trait Methylation Loci (eQTMs).** Linear regression (two-sided) was employed for this analysis.

File Name: Supplementary Data 21

Description: ***ASXL1* CHIP Expression Quantitative Trait Methylation Loci (eQTMs).** Linear regression (two-sided) was employed for this analysis.

File Name: Supplementary Data 22

Description: **Any CHIP Expression Quantitative Trait Methylation Loci (eQTM) Gene Ontology Enrichment Analysis.** The missMethyl R package was used for GO enrichment analysis. Fisher's Exact Test (one-sided) was used for this analysis.

File Name: Supplementary Data 23

Description: ***DNMT3A* CHIP Expression Quantitative Trait Methylation Loci (eQTM) Gene Ontology Enrichment Analysis.** The missMethyl R package was used for GO enrichment analysis. Fisher's Exact Test (one-sided) was used for this analysis.

File Name: Supplementary Data 24

Description: ***TET2* CHIP Expression Quantitative Trait Methylation Loci (eQTM) Gene Ontology Enrichment Analysis.** The missMethyl R package was used for GO enrichment analysis. Fisher's Exact Test (one-sided) was used for this analysis.

File Name: Supplementary Data 25

Description: ***ASXL1* CHIP Expression Quantitative Trait Methylation Loci (eQTM) Gene Ontology Enrichment Analysis.** The missMethyl R package was used for GO enrichment analysis. Fisher's Exact Test (one-sided) was used for this analysis.

File Name: Supplementary Data 26

Description: **CpGs Linked to *cis*-mQTLs with GWAS Catalog Traits for any CHIP and CHIP Subtypes.**

File Name: Supplementary Data 27

Description: **CpGs Linked to EWAS Catalog Traits for any CHIP and CHIP Subtypes.** Significance was defined at $0.05/4023 = 1.24 \times 10^{-5}$, based on one-sided P-values from an inverse variance-weighted fixed-effect meta-analysis.

File Name: Supplementary Data 28

Description: **Two-Sample Mendelian Randomization.** A two-sample Mendelian Randomization (MR) analysis was performed.

File Name: Supplementary Data 29

Description: **GO Enrichment Analysis for Genes Annotated to TET2-associated CpGs with Decreased Methylation.** The missMethyl R package was used for GO enrichment analysis. Fisher's Exact Test (over-representation one-sided) was used for this analysis.
