# Supplementary material for: Epigenome-wide DNA methylation association study of CHIP provides insight into perturbed gene regulation
Source: Nat Commun. 2025 May 20;16:4678. doi: 10.1038/s41467-025-59333-w (PMC12092741; doi:10.1038/s41467-025-59333-w)
Supplement: Supplementary file 32 — Reporting Summary [file 41467_2025_59333_MOESM32_ESM.pdf]

nature portfolio

Corresponding author(s): Daniel Levy

Last updated by author(s): Feb 26, 2025

Reporting Summary

Nature Portfolio wishes to improve the reproducibility of the work that we publish. This form provides structure for consistency and transparency in reporting. For further information on Nature Portfolio policies, see our Editorial Policies and the Editorial Policy Checklist.

Statistics

For all statistical analyses, confirm that the following items are present in the figure legend, table legend, main text, or Methods section.

n/a	Confirmed	
☐	☒	The exact sample size (*n*) for each experimental group/condition, given as a discrete number and unit of measurement
☐	☒	A statement on whether measurements were taken from distinct samples or whether the same sample was measured repeatedly
☐	☒	The statistical test(s) used AND whether they are one- or two-sided<br>*Only common tests should be described solely by name; describe more complex techniques in the Methods section.*
☐	☒	A description of all covariates tested
☐	☒	A description of any assumptions or corrections, such as tests of normality and adjustment for multiple comparisons
☐	☒	A full description of the statistical parameters including central tendency (e.g. means) or other basic estimates (e.g. regression coefficient) AND variation (e.g. standard deviation) or associated estimates of uncertainty (e.g. confidence intervals)
☐	☒	For null hypothesis testing, the test statistic (e.g. *F*, *t*, *r*) with confidence intervals, effect sizes, degrees of freedom and *P* value noted<br>*Give P values as exact values whenever suitable.*
☒	☐	For Bayesian analysis, information on the choice of priors and Markov chain Monte Carlo settings
☒	☐	For hierarchical and complex designs, identification of the appropriate level for tests and full reporting of outcomes
☐	☒	Estimates of effect sizes (e.g. Cohen's *d*, Pearson's *r*), indicating how they were calculated

Our web collection on statistics for biologists contains articles on many of the points above.

Software and code

Policy information about availability of computer code

Data collection	CHIP calls were used the Mutect2 software. The details of CHIP calls pipeline were in https://app.terra.bio/#workspaces/terra-outreach/CHIP-Detection-Mutect.<br>DNA methylation data from FHS, JHS, CHS, and ARIC were normalized and processed using R packages described in the Supplementary Methods, including watermelon, COMBat, surrogate variable analysis (SVA), and the Houseman method.<br>DNA methylation data for hematopoietic stem cells were processed using Biomodal pipeline (version 1.1.1) and cutadapt.
Data analysis	EWAS and Meta-analysis: Conducted using the lm(), lme(), and metagen() functions in R.<br>GO Enrichment Analysis: Performed using the missMethyl R package and the DAVID Bioinformatics online tool (https://david.ncifcrf.gov/home.jsp).<br>Mendelian Randomization (MR) Analysis: Conducted using our in-house analytical pipeline, MR-Seek (https://github.com/OpenOmics/mr-seek.git).<br>The scripts for generated figures were provided in Source data.

For manuscripts utilizing custom algorithms or software that are central to the research but not yet described in published literature, software must be made available to editors and reviewers. We strongly encourage code deposition in a community repository (e.g. GitHub). See the Nature Portfolio guidelines for submitting code & software for further information.

Data

Policy information about availability of data

All manuscripts must include a data availability statement. This statement should provide the following information, where applicable:

- Accession codes, unique identifiers, or web links for publicly available datasets
- A description of any restrictions on data availability
- For clinical datasets or third party data, please ensure that the statement adheres to our policy

The whole genome sequencing (WGS), DNA methylation, RNA sequencing data, and phenotypic data from the Framingham Heart Study (FHS), the Jackson Heart Study (JHS), the Cardiovascular Health Study (CHS), and the Atherosclerosis Risk in Communities (ARIC) study have been deposited in the dbGaP database [https://www.ncbi.nlm.nih.gov/gap/] under accession codes, phs000007.v32.p13 (FHS), phs000964 (JHS), phs001368 (CHS) phs000668 (ARIC). The WGS, DNA methylation, and phenotypic data from all cohorts used in this study are available under restricted access to protect participant privacy and ensure confidentiality. Access can be obtained by submitting an ancillary study proposal and obtaining IRB approval. Timelines for the approval process range from 4–9 weeks for CHS and 3–6 weeks for ARIC ancillary studies, with specific criteria and proposal forms for the respective studies available at https://chs-nhlbi.org/node/6222 and https://sites.cscc.unc.edu/aric/ancillary-studies-pfg. Source data are provided with this paper.

Research involving human participants, their data, or biological material

Policy information about studies with human participants or human data. See also policy information about sex, gender (identity/presentation), and sexual orientation and race, ethnicity and racism.

Reporting on sex and gender	Sex (biological attribute) was used in the project. Sex was self-reported by participants. Sex was used as a covariate in the linear mixed models testing associations between CHIP status and DNA methylation.
Reporting on race, ethnicity, or other socially relevant groupings	Race was self-reported by participants and used in the race-stratified analyses. We performed these analyses to see if stratification by race, which may capture different social and environmental exposures, impacts the epigenome. More information about this is reported in the Meta-analysis section of the Methods.
Population characteristics	The mean ages during whole genome sequencing for participants were 57, 56, and 58 in Framingham Heart Study (FHS), Jackson Heart Study (JHS), and Atherosclerosis Risk in Communities (ARIC) cohorts, respectively. Participants from the CHS cohort were older, with a mean age of 74. All cohorts had more women than men (54-63%). CHIP mutations with a variant allele frequency (VAF)) ≥2% were present in 5% (166/3295) of participants in FHS, 4% (68/1664) in JHS, 5% (142/2655) in ARIC, and 15% (86/582) in CHS. The full population characters are provided in Table 1 of the manuscript.
Recruitment	For the Original Cohort, FHS investigators sent letters based on random sampling of 2 of every 3 families with individuals aged 30-59, living in Framingham, MA. The subsequent cohorts - Offspring and Third Generation - include participants from the next generation of the Original and the Offspring cohort, respectively.<br><br>Participants in JHS were recruited among African-American adults from urban and rural areas of three counties (Hinds, Madison, and Rankin) of Jackson, MS, metropolitan statistical area (MSA). Participants were enrolled from each of these four recruitment pools: random (17%), volunteer (30%), currently enrolled in the ARIC Study (31%), and secondary family members (22%). Participant recruitment was limited to non-institutionalized adult African Americans 35-84 years old, except in the family cohort where participants of 21-34 years of age were eligible.<br><br>CHS recruited 5,888 participants who were aged 65 or older in four U.S. communities - Sacramento, CA; Hagerstown, MD; Winston-Salem, NC; and Pittsburgh, PA. Participants were sampled from the Health Care Financing Administration's (HCFA) Medicare eligibility lists.<br><br>The ARIC study consists of 15,792 men and women between 45-64 who were selected at random and recruited from the four following U.S. study communities between 1986 and 1989: Forsyth County, North Carolina, Jackson, Mississippi, Eight northern suburbs of Minneapolis, Minnesota, and Washington County, Maryland.<br><br>Several biases may present in these studies, including self-selection and geographic bias. There may be self-selection bias as participants who choose to join these studies may be more health-conscious than the general population - potentially skewing results. The recruitment of participants from specific regions like Framingham, MA may limit the generalizability of findings to other populations. Additionally, studies like the FHS which have primarily White participants have limited applicability of their findings to other racial/ethnic groups.
Ethics oversight	All participants provided written, informed consent. The study protocol was approved by the following institutional review boards at each collaborating institution: Institutional Review Board at Boston Medical Center (FHS); University of Washington Institutional Review Board (CHS); University of Mississippi Medical Center Institutional Review Board (ARIC: Jackson Field Center); Wake Forest University Health Sciences Institutional Review Board (ARIC: Forsyth County Field Center); University of Minnesota Institutional Review Board (ARIC: Minnesota Field Center); Johns Hopkins University School of Public Health Institutional Review Board (ARIC: Washington County Field Center); University of Mississippi Medical Center (JHS); Jackson State University (JHS); and Tougaloo College (JHS). All research was performed in accordance with relevant ethical guidelines and regulations. The study design and conduct adhered to all relevant regulations regarding the use of human study participants and was conducted in accordance to the criteria set by the Declaration of Helsinki.

Note that full information on the approval of the study protocol must also be provided in the manuscript.

Field-specific reporting

Please select the one below that is the best fit for your research. If you are not sure, read the appropriate sections before making your selection.

☒ Life sciences ☐ Behavioural & social sciences ☐ Ecological, evolutionary & environmental sciences

For a reference copy of the document with all sections, see nature.com/documents/nr-reporting-summary-flat.pdf

Life sciences study design

All studies must disclose on these points even when the disclosure is negative.

Sample size	This study used all available samples (n=8196) in FHS whose DNA methylation data and CHIP calling (passing quality control) were both available.
Data exclusions	We excluded samples whose DNA methylation data failed quality control as provided in Supplementary File 2.
Replication	The multiracial meta-EWAS methylation findings were successfully replicated in CRISPR/CAS9-edited human CHIP cells. Flow sorted cell populations were used to ensure a more rigorous metric to define the cell population that is being assessed.
Randomization	This is not relevant to our study as observational, community-based cohorts were used.
Blinding	This is not relevant to our study as observational, community-based cohorts were used.

Reporting for specific materials, systems and methods

We require information from authors about some types of materials, experimental systems and methods used in many studies. Here, indicate whether each material, system or method listed is relevant to your study. If you are not sure if a list item applies to your research, read the appropriate section before selecting a response.

Materials & experimental systems

n/a	Involved in the study	
☐	☒	Antibodies
☐	☒	Eukaryotic cell lines
☒	☐	Palaeontology and archaeology
☒	☐	Animals and other organisms
☒	☐	Clinical data
☒	☐	Dual use research of concern
☒	☐	Plants

Methods

n/a	Involved in the study	
☒	☐	ChIP-seq
☐	☒	Flow cytometry
☒	☐	MRI-based neuroimaging

Antibodies

Antibodies used	CD34-APC/Cy7 RRID: AB_2571927 (Biolegend: 343614) dilution 1:50<br>CD38-BV605 RRID: AB_2562915 (Biolegend 303532) dilution 1:100<br>Lineage Cocktail-Pacific Blue RRID: AB_2889063(Biolegend 348805) dilution 1:10
Validation	CD34-APC/Cy7; Anti-Human; Host Species Mouse; Citations: Maury, E. et al Nat Commun 2021, Hua J, et al. 2023. Ann Transl Med. 11:62., Hinterbrandner M, et al. 2021. JCI Insight. 6:e151797<br><br>CD38-BV605; Anti-Human; Host Species Mouse; Citations: Vanuytsel K, et al. 2022. Cytometry A. 101:903., Swadling L, et al. 2020. Cell Rep. 30:687., Watts J, et al. 2022. Front Oncol. 11:806243.<br><br>Lineage Cocktail-Pacific Blue; Anti-Human; Host Species Mouse; Citations: Wang X, et al. 2021. Cell Discovery. 7(1):60., Hu EY, et al. 2020. JCI Insight. 5:00, Schielke L, et al. 2022. Front Immunol. 13:916701., Moravcikova E, et al. 2018. Cytometry A. 93:894., Saygin C, et al. 2021. Leukemia. 35:3406., Verma M, et al. 2021. J Exp Med. 218:.

Eukaryotic cell lines

Policy information about cell lines and Sex and Gender in Research

Cell line source(s)	Cell line Source(s):<br>mPB-001: Sex: Female; Supplier Fred Hutchinson<br>mPB-002: Sex: Male; Supplier Fred Hutchinson

mPB-003: Sex: Female; Supplier StemCell Technologies
mPB-004: Sex: Male; Supplier: StemCell Technologies
mPB-005: Sex: Male; Supplier: StemCell Technologies

Authentication

Flow Cytometry was performed by supplier.

Mycoplasma contamination

The cell lines were not tested for mycoplasma contamination.

Commonly misidentified lines
(See ICLAC register)

No commonly misidentified cell lines were used during this study.

Plants

Seed stocks

Report on the source of all seed stocks or other plant material used. If applicable, state the seed stock centre and catalogue number. If plant specimens were collected from the field, describe the collection location, date and sampling procedures.

Novel plant genotypes

Describe the methods by which all novel plant genotypes were produced. This includes those generated by transgenic approaches, gene editing, chemical/radiation-based mutagenesis and hybridization. For transgenic lines, describe the transformation method, the number of independent lines analyzed and the generation upon which experiments were performed. For gene-edited lines, describe the editor used, the endogenous sequence targeted for editing, the targeting guide RNA sequence (if applicable) and how the editor was applied.

Authentication

Describe any authentication procedures for each seed stock used or novel genotype generated. Describe any experiments used to assess the effect of a mutation and, where applicable, how potential secondary effects (e.g. second site T-DNA insertions, mosiacism, off-target gene editing) were examined.

Flow Cytometry

Plots

Confirm that:

☒ The axis labels state the marker and fluorochrome used (e.g. CD4-FITC).

☒ The axis scales are clearly visible. Include numbers along axes only for bottom left plot of group (a 'group' is an analysis of identical markers).

☒ All plots are contour plots with outliers or pseudocolor plots.

☒ A numerical value for number of cells or percentage (with statistics) is provided.

Methodology

Sample preparation

Samples are mobilized peripheral blood CD34+ cells isolated by supplier (StemCell Technologies or Fred Hutchinson). Samples were thawed and cultured for a total of 9 days (day 2 were edited with CRISPR-Cas9 and flow sorted on Day 9). Samples were washed with cell staining buffer from Biolegend. Then cells were stained with Live/Dead stain(7-AAD) and antibodies targeting CD34+, CD38+, and Lineage cocktail(CD3,CD14,CD16,CD19,CD20,CD56).

Instrument

BD FACSymphony S6 Cell Sorter

Software

All data was processed and analyzed using FlowJo version 10.

Cell population abundance

97-100% Purity of Live CD34+(POS) CD38-(NEG) Lineage-(NEG) cells. The signal for the sorted population was compared the signal to an unstained control.

Gating strategy

Unstained controls were used to determine background fluorescence followed by a live/dead stain only control for targeting live cells. All remaining single stain controls for each antibody were determined with beads for marking positive/negative gates. This experimental design strategy was used for each flow experiment. The gating strategy was as follows: preliminary forward and side scatter gates were made on the cells followed by removal of doublets with SSC-A vs SSC-H and FSC-A vs FSC-H. Live cells were then selected by removal of positive stain towards 7-AAD(negative stain = Live cells). Gates were then made for samples, which had differentiated as seen by a cocktail of lineage markers (CD3, CD14, CD16, CD19, CD20, CD56) these cells were removed, and the final gate was on CD34 and CD38 where CD34+CD38- cells were sorted into a collection tube.

☒ Tick this box to confirm that a figure exemplifying the gating strategy is provided in the Supplementary Information.
